# Recent development of allele frequencies and exclusion probabilities of microsatellites used for parentage control in the German Holstein Friesian cattle population

**DOI:** 10.1186/s12863-016-0327-z

**Published:** 2016-01-08

**Authors:** Bertram Brenig, Ekkehard Schütz

**Affiliations:** Institute of Veterinary Medicine, Georg-August-University Göttingen, Burckhardtweg 2, D-37077 Göttingen, Germany

**Keywords:** Parentage control, Cattle, Holstein Friesian, Microsatellite, Single nucleotide polymorphism, Allele frequency, Exclusion probability, Hitchhiking

## Abstract

**Background:**

Methods for parentage control in cattle have changed since their initial implementation in the late 1950’s from blood group typing to more current single nucleotide polymorphism determination. In the early 1990’s, 12 microsatellites were selected by the International Society for Animal Genetics based on their informativeness and robustness in a variety of different cattle breeds. Since then this panel is used as standard in cattle herd book breeding and its application is accompanied by recurrent international comparison tests ensuring permanent validity for the most common commercial dairy and beef cattle breeds for example Holstein Friesian, Simmental, Angus, and Hereford. Although, nearly every parentage can be resolved using these microsatellites, cases with very close relatives became an emerging resolution problem during recent years. This is mainly due to an increase of monomorphism and a trend to the fixation of alleles, although no direct selection against their variability was applied. Thus other effects must be presumed resulting in a loss of polymorphism information content, heterozygosity, and exclusion probabilities.

**Results:**

To determine changes of allele frequencies and exclusion probabilities, we analyzed the development of these parameters for the 12 microsatellites from 2004 to 2014. One hundred sixty eight thousand recorded Holstein Friesian cattle genotypes were evaluated. During this period certain alleles of nine microsatellites increased significantly (t-values >5). When calculating the exclusion probabilities for 11 microsatellites, reduction was determined for the three situations, i.e. one parent is wrongly identified (*p* = 0.01), both parents are wrongly identified (*p* = 0.005), and the genotype of one parent is missing (*p* = 0.048). With the addition of BM1818 to the marker set in 2009, this development was corrected leading to significant increases in exclusion probabilities. Although, the exclusion probabilities for the three family situations using the 12 microsatellites are >99 %, the clarification of 142 relationships in 40,000 situations where one parent is missing will still be impossible.

Twenty-five sires were identified that are responsible for the most significant microsatellite allele increases in the population. The corresponding alleles are mainly associated with milk protein and fat yield, body weight at birth and weaning, as well as somatic cell score, milk fat percentage, and longissimus muscle area.

**Conclusions:**

Our data show that most of the microsatellites used for parentage control in cattle show directional changes in allele frequencies consistent with the history of artificial selection in the German Holstein population.

## Background

Parentage control and traceability is an important issue in animal production and usually obligatory for animals used in breeding programs [1–6]. For routine parentage diagnosis, analyses should fulfill a variety of technical requirements, for instance easy handling, robustness, reproducibility, standardization, possibility of automation, short processing time, and reasonable costs. However, the most important prerequisite for markers used in parentage control is the ability to discriminate between even very close relatives [3]. Therefore, during the last seven decades methods for parentage control in cattle have changed considerably. In the early 1940’s and late 1950’s cattle blood groups were identified and shown to be useful in parentage control [7–10]. However, due to intensive inbreeding in the Holstein Friesian population and limited variability, blood groups became increasingly uninformative over time. Hence, approximately 40 years later, with the rapid development of molecular biological techniques and genome data, blood group typing was replaced by the use of highly polymorphic DNA markers, so-called mini- and microsatellites. The use of minisatellites in DNA fingerprinting and identification of individuals was first described in humans [11, 12], rapidly also entering the area of domestic animal identification and pedigree analysis [13]. Initial steps in using mini- and microsatellites in cattle identification and parentage control were done only a few years later [14, 15] and further actions were taken to establish a robust and internationally comparable panel of markers [16–20]. In international comparison tests under the direction of the International Society for Animal Genetics (ISAG) a panel of at least 12 microsatellite markers (short tandem repeats-STR) was established for parentage control in cattle. The 12 markers comprised BM1814, BM1818, BM2113, ETH3, ETH10, ETH225, INRA023, SPS115, TGLA53, TGLA122, TGLA126, TGLA227 [21–27]. Since the mid 1990’s this panel is used worldwide for parentage control and after approximately 15 years this is now in the process of being replaced again by the use of 100 and/or 200 single nucleotide polymorphisms (SNP). These SNPs are a sub-set taken from markers used for genomic selection or genome wide association analysis [28–30].

However, microsatellites are still the gold standard for parentage control in most breeding programs of beef as well as dairy cattle, based on the ease of testing, testing availability and million of results in the breeding databases. In this context it is important to review continuously the genetic variability and exclusion probabilities of the applied microsatellites [17, 19]. Ideally, microsatellites should be neutral DNA markers maintaining their characteristics relatively constant. Neutral DNA markers are solely subjected to stochastic processes such as mutation and genetic drift [31]. However, several of the microsatellites in the ISAG parentage control panel are under artificial selection and therefore actually not completely neutral. ETH10 on bovine chromosome 5 for example seems to be associated with growth and carcass traits in Angus, Brangus, and other cattle breeds [32, 33]. The ETH10 locus was also associated with coat colour in a Charolais x Holstein resource population and arachnomelia in Brown Swiss cattle [34, 35]. BM1818 was shown to be associated with somatic cell score (SCS) and specific alleles of this microsatellite are either favourable or unfavourable for mastitis resistance [36]. In another study, significant differences in allelic frequencies for BM1824, ETH10, INRA023, SPS115 and TGLA53 alleles were described in lines of Japanese Black cattle depending on selection of sires for intramascular fat [37]. It must therefore be assumed that due to selection for specific traits the variability and exclusion probabilities will decrease. As a consequence, the microsatellite panel will become increasingly uninformative especially in situations where very close relatives have to be tested. This seems in particular foreseeable in livestock with an active and well established breeding program, where certain sires can become predominant, if their breeding value is exceptional. To prove this hypothesis, we have evaluated the development in allele distribution of the internationally used STR markers in the German Holstein population over the last decade.

## Results

Microsatellite genotypes of the German Holstein Friesian population (GHF) were analyzed, generated in the frame of routine parentage control using the standardized microsatellite panel recommended by ISAG. From 2004 to 2008, no data were available for BM1818, which was added to the panel only in 2009. Table 1 shows the number and lengths of alleles (standardized according to animal No. 13 of the ISAG cattle comparison test 2005) detected for each microsatellite marker in GHF together with the respective repeat numbers that have been determined by sequencing elsewhere [38]. For microsatellites BM1818, BM2113, ETH3 and TGLA227 several alleles that have been described previously were not detected in the GHF. On the other hand, a larger number of markers i.e. BM1824, ETH10, ETH225, INRA023, SPS115, TGLA122, TGLA126, TGLA227 and TGLA53, showed alleles that have not yet been described.

**Table 1:** Microsatellites alleles (bp) detected in the Holstein Friesian population

	Microsatellite allele (bp)^a^											
Repeat number	1^b^	2	3	4	5	6	7	8	9	10	11	12
9											*71*	
10		*174* ^d^					*192*					
11							*194*				**75**	
12		178	121				*196*			*105*	77	
13	**256** ^c^	180	**123**				198			*107*	79	
14	258	182	125		209		200		137	109	81	
15	260	184	127	**103**	*211*		202		139	111	83	
16	262	*186*	129		213		204		141	113	85	
17	264	188	131		215		206	*240*	143	115	87	*150*
18	266	190	133	109	217		208		145	117	89	*152*
19	268		135		219	140	210	*244*	147	119	91	154
20	270		137		221	142	212	*246*	149	121	93	156
21	**272**		139	115	223	144	214	248	151	123	95	158
22			141	117	225	146	216	250	153	125	97	160
23			143	119		148	218	252	155		99	162
24				121		150	220	254	157		101	164
25	**280**			123		152	222	256	159		103	166
26				125		*154*		258	161			168
27				127		*156*		260	163			170
28				129		*158*		262	165			172
29				131		*160*			167			174
30									169			176
31									171			178
32									173			180
33									175			182
34									177			184
35									179			186
36									181			188
37									183			190
38									*185*			*192*
39												
40												*196*

^a^Allele sizes are standardized according animal No. 13 of the ISAG cattle comparison test 2005. ^b^1: BM1818, 2: BM1824, 3: BM2113, 4: ETH3, 5: ETH10, 6: ETH225, 7: INRA023, 8: SPS115, 9: TGLA122, 10: TGLA126, 11: TGLA227, 12: TGLA53. ^c^Alleles that have been described, but are not present in the HF population are shown in bold. ^d^Alleles that have not been described, but are present in the HF population are italicized

**Fig. 1:**
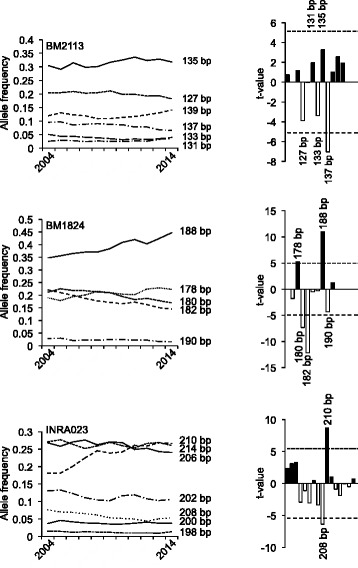
Development of allele frequencies of microsatellite markers BM2113, BM1824, and INRA023 from 2004 to 2014. The *left side* of the figure illustrates the development of the major alleles of the respective microsatellite markers from 2004 to 2014. The significance of the increases or decreases in allele frequencies was calculated as described above and is shown on the *right side*. Sizes of alleles with t-values >5 are indicated. *Black bars* correspond to alleles with increased, *open bars* correspond to alleles with decreased allele frequencies

**Fig. 2:**
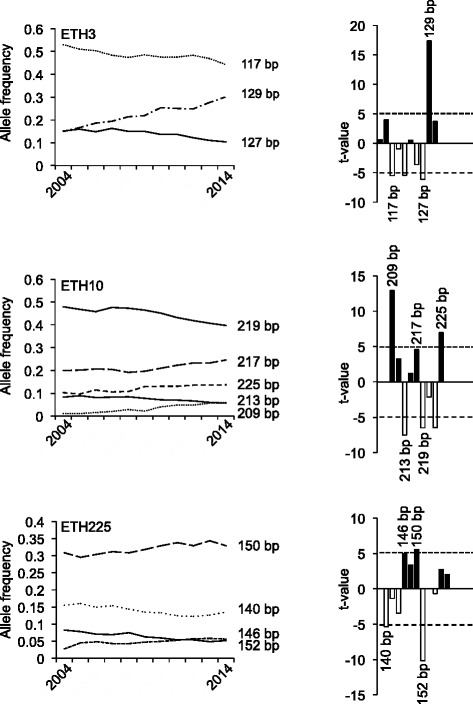
Development of allele frequencies of microsatellite markers ETH3, ETH10, and ETH225 from 2004 to 2014. The *left side* of the figure illustrates the development of the major alleles of the respective microsatellite markers from 2004 to 2014. The significance of the increases or decreases in allele frequencies was calculated as described above and is shown on the *right side*. Sizes of alleles with t-values >5 are indicated. *Black bars* correspond to alleles with increased, *open bars* correspond to alleles with decreased allele frequencies

**Fig. 3:**
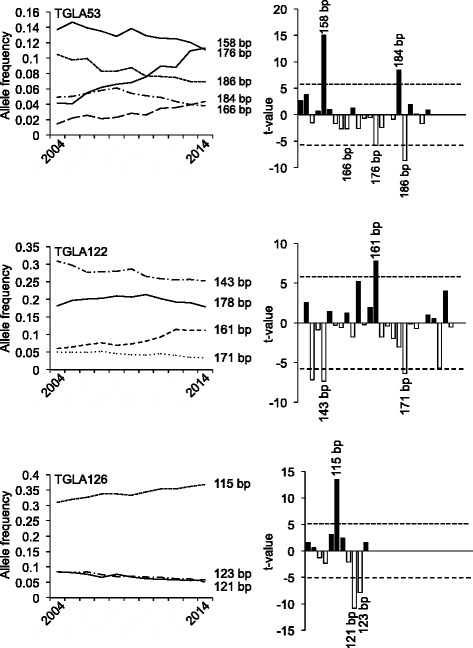
Development of allele frequencies of microsatellite markers TGLA53, TGLA122, and TGLA126 from 2004 to 2014. The *left side* of the figure illustrates the development of the major alleles of the respective microsatellite markers from 2004 to 2014. The significance of the increases or decreases in allele frequencies was calculated as described above and is shown on the *right side*. Sizes of alleles with t-values >5 are indicated. *Black bars* correspond to alleles with increased, *open bars* correspond to alleles with decreased allele frequencies

**Fig. 4:**
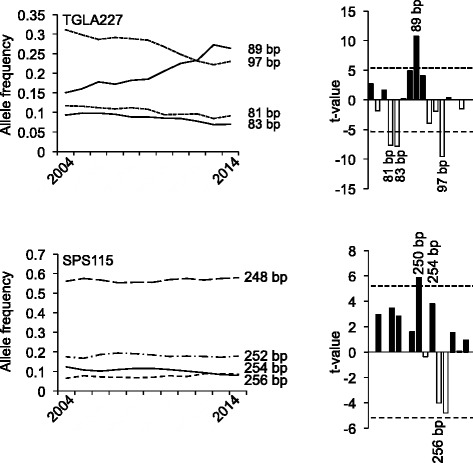
Development of allele frequencies of microsatellite markers TGLA227 and SPS115 from 2004 to 2014. The *left side* of the figure illustrates the development of the major alleles of the respective microsatellite markers from 2004 to 2014. The significance of the increases or decreases in allele frequencies was calculated as described above and is shown on the *right side*. Sizes of alleles with t-values >5 are indicated. *Black bars* correspond to alleles with increased, *open bars* correspond to alleles with decreased allele frequencies

Allele frequencies for all markers that were calculated for each year and alleles with significant changes in frequencies during the 11 years are shown in Figs. 1, 2, 3 and 4. Those alleles showing a significant trend during the observation period are summarized in Table 2. All microsatellites showed either increases or decreases of specific alleles during the analyzed period. Only BM2113, showed no significant frequency increase of a single allele. For all other markers at least one allele increased significantly over the evaluated 11 years period.

**Table 2:** Development of microsatellite allele frequencies from 2004 to 2014

STR^a^	Repeat number	Allele (bp)	f_(2014)_^b^	t-value^c^	t-value^d^
BM1824	17	188	0.446	10.99	8.73
	12	178	0.223	5.25	3.85
ETH3	28	129	0.301	17.38	15.05
ETH10	14	209	0.059	12.9	11.62
	22	225	0.138	6.97	5.62
ETH225	24	150	0.329	5.57	3.68
INRA023	19	210	0.267	8.67	7.23
SPS115	22	250	0.002	5.86	5.28
TGLA53	21	158	0.113	15.07	13.31
	34	184	0.044	8.42	3.47
TGLA122	26	161	0.112	7.79	6.72
TGLA126	17	115	0.681	13.45	9.65
TGLA227	18	89	0.264	10.75	9.23
BM1824	13	180	0.169	−7.34	−5.58
	14	182	0.145	−12.17	−9.65
BM2113	20	137	0.066	−7.04	−5.38
ETH3	22	117	0.443	−5.04	−3.95
	24	121	0.009	−5.43	−4.29
	27	127	0.104	−6.16	−4.89
ETH10	16	213	0.056	−7.53	−5.96
	19	219	0.397	−6.50	−4.94
	21	223	0.047	−6.45	−5.07
ETH225	19	140	0.135	−5.36	−3.93
	25	152	0.052	−10.18	−8.16
INRA023	18	208	0.053	−6.37	−5.04
TGLA53	30	176	0.111	−5.84	−4.10
	35	186	0.069	−8.65	−3.56
TGLA122	15	139	0.001	−7.16	−6.14
	17	143	0.252	−7.35	−5.23
	31	171	0.034	−6.37	−4.92
TGLA126	20	121	0.051	−10.84	−8.57
	21	123	0.058	−7.85	−6.14
TGLA227	14	81	0.092	−7.68	−5.78
	15	83	0.069	−7.83	−6.06
	22	97	0.231	−9.57	−7.62

^a^ *STR* short tandem repeat, ^b^ *f*_*(2014)*_ Allele frequency at the end of 2014. ^c^0-hypothesis: slope = 0; ^d^vs expected maximal random change

Those detected significant linear trends were compared to the theoretic values that could occur by random genetic drifts. For this, the expected development of allele frequencies in a random population over 10 years with an effective population size of 103 [39] was assessed and compared to the observed trends.

For all STR alleles that show a highly significant trend vs. a zero-slope, the significance was still <0.05 when compared to the maximum expected random slope (Table 2).

**Fig. 5:**
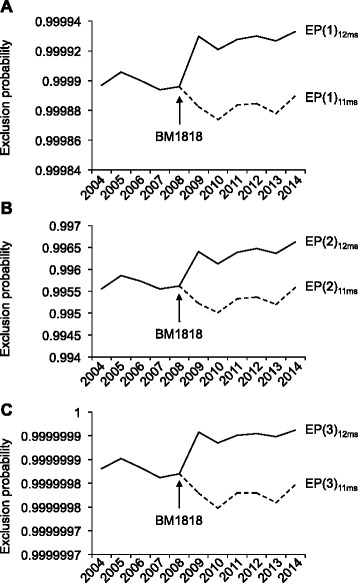
Development of the exclusion probabilities using 11 or 12 microsatellite markers from 2004 to 2014. Exclusion probabilities were calculated for the three family situations [40], (**a**) two parents and one offspring is given; exclude a parent, (**b**) one parent and one offspring is given; exclude their relationship, (**c**) two parents and one offspring are given; exclude both parents. The addition of marker BM1818 in 2009 is indicated with an *arrow*

To analyze, whether the changes in allele frequencies had an influence on the informativeness of the marker panel, we calculated the exclusion probabilities (EP) as previously described [40]. In Fig. 5 the development of the EPs are shown. In three situations the EPs displayed a similar course over the years with a reduction in exclusion probabilities when using only the initially recommended 11 microsatellites. With the addition of BM1818 in 2009 the maker panel reached an acceptable level of EP again.

In an approach to define the founder(s) of the detected changes in STR frequencies, we searched our database for all sires harboring alleles with the highest positive t-value, which are BM1824 (188 bp), ETH3 (129 bp), ETH10 (209 bp), INRA023 (210 bp), TGLA53 (158 bp), TGLA126 (115 bp) and TGLA227 (89 bp), and identified a total of 193 sires. The search criteria were then refined in a following step by the addition of the second most significantly increased alleles, i.e. ETH10 (225 bp), ETH225 (146 bp), SPS115 (250 bp), TGLA53 (184 bp), TGLA122 (161 bp) and BM1818 (268 bp). In addition alleles were included that showed an increasing tendency, i.e. BM2113 (135 bp), SPS115 (240 bp) and TGLA122 (183 bp). With these two refinements, the number of sires was reduced to 25 individuals.

## Discussion

For almost two decades, microsatellites have been used in parentage control in cattle breeding under the assumption that these markers remain informative even under intensive inbreeding and selection, as being trait neutral. Currently even though still relatively rare, in recent years problems arose in parentage control whenever ancestries of closely related animals had to be determined. Hence, although microsatellites used for parentage control should be neutral DNA markers, the data presented here clearly show that this is not the case. This is supported by earlier analyses describing that ETH10, BM1818, BM1824, INRA023, SPS115 and TGLA53 are somehow associated with different economical important traits [32–37].

The data presented here also indicate that ETH3, ETH225, TGLA122, TGLA126 and TGLA227 are most likely influenced either by selective breeding or by hitchhiking effect. Although BM2113 did not show frequency increase of any single allele, the significant reduction of the allele with 137 bp in GHF (Fig. 1) might indicate an unfavourable effect of this allele in breeding. This can also be assumed for significantly reduced alleles of the other microsatellites in the population shown in Table 2.

To analyze whether the increase or decrease in allele frequencies was due to the fact that most of the microsatellite markers are associated with economical important traits, we looked for QTLs at the chromosomal locations of the microsatellites. At least 40 different QTLs have been described flanking the microsatellite chromosomal positions and the most frequent traits included milk protein yield, milk fat yield, somatic cell score, milk fat percentage, body weight at birth and body weight at weaning [41]. From this it can be hypothesized that the allele frequency changes over the last several years are–at least in part–a consequence of selection for these traits.

According to the German Holstein Friesian Association, 1.61 million HF cows were registered in 2012 in Germany [42]. If an estimated effective population size of 103 is assumed [39], the number of males (*N*_*m*_) can be calculated to be 25.75 [43]. As this number seems to be rather low at a first sight, we wanted to see, whether a similar number would be obtained, when searching the GHF population for sires transmitting the most significantly increased alleles over the last decade (Figs. 1, 2, 3 and 4). Interestingly, this number agrees perfectly to the estimated number of males calculated from the effective population size. With the reverse search using the allele frequencies several famous HF sires were identified, e.g. Goldwin, Shottle, Hayden, Atwood, and Laudan.

Finally, we wanted to proof that the changes in allele frequencies are not due to random genetic drift. Therefore, we compared the expected development of allele frequencies in a random with the observed development in the GHF. The slopes calculated for both scenarios (genetic drift or hitchhiking) are significantly different and hence the process influencing the changes in allele frequencies is not only due to genetic drift.

## Conclusions

In summary, we were able to show that the microsatellite markers recommended for parentage control in cattle are influenced by selective breeding and are therefore adaptive DNA markers. Nearly all of the microsatellites are located in QTL regions or are associated with genes influencing the breeding value. Consequently, during the last 10 years several alleles significantly increased or decreased in frequency resulting in a reduced overall informativeness and exclusion power of the marker panel. This problem can only be solved by the inclusion of additional markers to the panel. Similar recommendations can be given for the foreseeable exclusive use of SNPs in the near future. The evaluation and applicability of SNPs in parentage control has been shown in several studies [3, 30, 44–46]. However, it is also clear that the currently recommended minimal number of SNPs might not be sufficient to eliminate false-negative results [28, 47].

## Methods

### Ethical statement

Data are based on rotuine diagnostic parentage control performed with written owner consent. Collection of blood samples was conducted exclusively by local veterinarians. Blood sampling by veterinarians with state examination is in accordance with the German Animal Welfare Act (§6 Abs. 1 Satz 2 TierSchG). Therefore no formal ethical approval was required, since no other samples were collected for this study.

### DNA samples and genotyping

A total of 168,000 Holstein Friesian cattle were genotyped. DNA from blood samples was extracted using a salting out procedure [48] or the MagNA Pure LC DNA Isolation Kit I (Roche Diagnostics). For the isolation of DNA from tissue/hair samples the DNeasy Blood and Tissue Kit (Qiagen) or the MagNA Pure LC DNA Isolation Kit II (Roche Diagnostics) was used according to the manufacturer’s protocols.

For genotyping the StockMarks® for Cattle Genotyping Kit (Life Technologies™) or after cessation of that a laboratory developed multiplex method was used and allele sizes were adjusted to the reference animal No. 13 from the ISAG cattle comparison test 2005. Reactions were separated on an ABI PRISM® 3130xl Genetic Analyzer (Life Technologies™) according to the manufacturers’ protocols. DNA profiles were recorded with Data Collection v3.1.1 and evaluted using GeneMapper v4.1 (Life Technologies™). From the database, the allelic frequencies were calculated on a yearly basis over the period from 2004 to 2014.

### Statistical analysis

All statistical analyses were done with Microsoft® Excel® for Mac version 14.4.8 (150116). Exclusion probabilities were calculated as described previously [40]. The variance in allele frequency after t generations (*V*_*t*_) was calculated as described [49]. The effective population size (*N*_*e*_) of the German Holstein population was set to 103 in accordance to estimations based on linkage disequilibrium data published earlier [39].

For each of the STRs, a linear trend was assessed for any occurring allele using the least-square linear regression model. For statistical purposes, the usual definition of the regression t-value was used (slope/SE_slope_). Such a trend was considered significant if the calculated t-value (0-hypothesis as slope = 0) exceeded the corresponding *p*-value, considering the degrees of freedom (9) and after Bonferroni correction for the individual number of alleles for the STR. The calculation was performed against the maximum theoretical slope (in the same direction as the observed slope) based on *V*_*t*_, using the observed standard error of the slope as denominator ([slope_observed_ − slope_expected_]/SE_slope(obs)_).

## Abbreviations

EP: exclusion probability
GHF: German Holstein Friesian
HF: Holstein Friesian
ISAG: International Society for Animal Genetics
QTL: quantitative trait locus
SCS: somatic cell score
SNP: single nucleotide polymorphism
STR: short tandem repeat
